# In Situ Observation of Crystalline Silicon Growth from SiO_2_ at Atomic Scale

**DOI:** 10.34133/2019/3289247

**Published:** 2019-10-30

**Authors:** Kaihao Yu, Tao Xu, Xing Wu, Wen Wang, Hui Zhang, Qiubo Zhang, Luping Tang, Litao Sun

**Affiliations:** ^1^SEU-FEI Nano-Pico Center, Key Laboratory of MEMS of Ministry of Education, School of Electronic Science and Engineering, Southeast University, Nanjing 210096, China; ^2^Shanghai Key Laboratory of Multidimensional Information Processing, Department of Electronic Engineering, East China Normal University, Shanghai 200241, China

## Abstract

The growth of crystalline Si (c-Si) via direct electron beam writing shows promise for fabricating Si nanomaterials due to its ultrahigh resolution. However, to increase the writing speed is a major obstacle, due to the lack of systematic experimental explorations of the growth process and mechanisms. This paper reports a systematic experimental investigation of the beam-induced formation of c-Si nanoparticles (NPs) from amorphous SiO_2_ under a range of doses and temperatures by in situ transmission electron microscopy at the atomic scale. A three-orders-of-magnitude writing speed-up is identified under 80 keV irradiation at 600°C compared with 300 keV irradiation at room temperature. Detailed analysis reveals that the self-organization of c-Si NPs is driven by reduction of c-Si effective free energy under electron irradiation. This study provides new insights into the formation mechanisms of c-Si NPs during direct electron beam writing and suggests methods to improve the writing speed.

## 1. Introduction

With the development of semiconductor technology, fabrication of crystalline Si (c-Si) from amorphous SiO_2_ via direct electron beam writing is a promising method to fabricate Si-based nanodevices [1–3]. It is a one-step resistless process which avoids the resolution loss during development in a developer [4–8]. However, the writing speed is still the main handicap for practical applications due to only one pixel being exposed at a time [9, 10]. To improve the exposure speed is critical for direct electron beam writing. But it has been suggested that the writing current is limited by the Coulomb interaction between electrons, which causes beam blurring and loss of resolution [11].

Fully understanding the growth mechanisms of c-Si from SiO_2_ is essential for increasing exposure speed during direct writing. Du et al. obtained c-Si nanodots under 200 keV electron irradiation at ambient temperature with a dose of 1 × 10^8^ C m^−2^ and attributed the formation of amorphous Si to valence electron ionization and the subsequent transformation to c-Si to the elastic displacement of Si with a threshold beam energy of approximately 150.2 keV [12]. However, Takeguchi et al. grew c-Si under 100 keV electron irradiation at 577°C [13], and Chen et al. fabricated a Si nanodot array under 100 keV irradiation at room temperature with a dose of approximately 10^9^ C m^−2^ [14]. They believed that the Si nanodot formation mechanism is process-induced SiO_2_ dissociation. Hence, the mechanism of formation of c-Si under irradiation is still unclear, and the quantitative understanding of the influence of temperature and beam energy is very limited.

The interaction between high-energy electrons and SiO_2_ can be considered elastic and inelastic scattering. Elastic scattering is the interaction of an electron with an atomic nucleus that will induce atom displacement by direct momentum transfer if the transferred energy is larger than the threshold displacement energy (*T*_*d*_) [15]. The *T*_*d*_ of O and Si are 9.3 eV and 18.6 eV, respectively [16]; thus, the O atoms are more easily displaced. Inelastic scattering is the interaction of incident electrons with atomic electrons that can lead to ionization. The atomic electrons are considered valence electrons and core-shell electrons in SiO_2_. Ionization of a valence electron creates only one hole in the valence band with a lifetime of approximately 10^−16^ s, while ionization of a core-shell electron creates at least two holes in the valence band by the Knotek-Feibelman mechanism. The hole-hole correlations can block the resonant one-hole hopping process, thus increasing the lifetime of the holes to the order of 10^−14^ s. The presence of holes in the valence can induce a repulsive reaction between nearby nuclei and result in subsequent desorption of O. The desorption time for atoms is on the order of 10^−13^ s, so the core electron ionization has a larger probability of inducing dissociation of SiO_2_ [17].

In this work, we apply in situ transmission electron microscopy (TEM) to investigate the detailed growth process of c-Si nanoparticles (NPs) under different electron energies and temperatures to explore methods to increase the exposure speed during direct electron beam writing. In situ transmission electron microscopy is a powerful and versatile tool for real-time investigation of the properties of nanomaterials under electron irradiation and external stimulations [18–22]. We demonstrate that amorphous SiO_*x*_ (*x* < 2) NPs form firstly and then transform to c-Si NPs, when amorphous SiO_2_ is exposed to an electron shower. The elastic sputtering and the Knotek-Feibelman dissociation mechanisms induced desorption of O in SiO_2_, results in the formation of amorphous SiO_*x*_ NPs. The critical dose for SiO_*x*_ NP formation is independent of temperature and decreases with reducing electron beam energy. The formation of c-Si NPs is driven by the self-organization of Si atoms, which is caused by phase stability inversion between c-Si and amorphous SiO_*x*_ under electron irradiation. Detailed analysis reveals that a larger effective free energy difference between SiO_*x*_ and c-Si is critical to improve the speed during direct writing. This energy difference increases with decreasing electron beam energy and increasing temperature. The critical dose for c-Si NP formation can be decreased three orders of magnitude to approximately 10^5^ C m^−2^ under 80 keV irradiation at 600°C.

## 2. Results and Discussion

**Figure 1:**
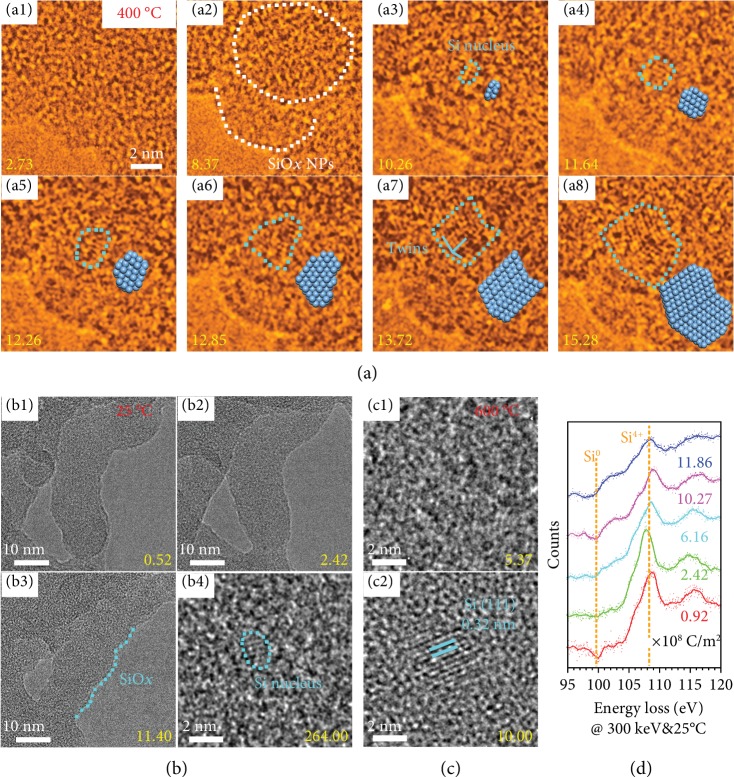
Dose and temperature dependence of Si nanocrystal formation under 300 keV electron irradiation. (a1–a8) False-color TEM images and atomic models show the dynamics of Si nanocrystal formation at 400°C. (a1) Amorphous SiO_2_. (a2) Formation of amorphous SiO_*x*_ NPs in SiO_2_. (a3–a8) The crystalline Si nanoparticle nucleates from an amorphous SiO_*x*_ nanoparticle and expends with an increase of the irradiation dose. (b1–b3) Formation process of SiO_*x*_ NPs at 25°C with an increase of the dosage. The initial rough surface becomes smooth at first, then amorphous SiO_*x*_ NPs appear at this surface, highlighted by the cyan dashed line. (b4) Crystalline Si nucleus in a SiO_*x*_ nanoparticle. (c1, c2) Growth process of c-Si NPs at 600°C under different dose rates. (d) EELS of Si *L*_2,3_ edge at 25°C under different doses, confirming the formation of elemental Si. The doses are shown by yellow numbers with the unit of 10^6^ C m^−2^.

Figure 1(a1)–1(a8) shows false-color TEM images of the c-Si NP formation dynamics under 300 keV electron irradiation at 400°C. Firstly, amorphous SiO_*x*_ NPs are observed when the irradiation dose is accumulating to 8.37 × 10^6^ C m^−2^, due to the desorption of O. The low magnification images in [Supplementary-material supplementary-material-1] (Supporting Information) clearly show the formation of nanoparticles in SiO_2_. Then, a Si nucleus is formed in the SiO_*x*_ NP at the dose of 1.026 × 10^7^ C m^−2^, shown in Figure 1(a3). Figure 1(a4)–1(a8) represents the growth process of the Si NP with an increase of irradiation dose. The growth of c-Si NPs is induced by attachment of Si atoms to the nucleus driven by free energy difference, which will be discussed latter. Due to this growth manner, once the misattachment of atoms occurs, the formation of twins will be observed (Figure 1(a8)). The c-Si NP size is about 4 nm and the dose is 1.528 × 10^7^ C m^−2^.

To investigate the influence of temperature, SiO_2_ is irradiated at 25°C and 600°C. Figure 1(b1)–1(c2) shows images of amorphous SiO_2_ after 300 keV electron irradiation at 25°C and 600°C. The images in Figure 1(b1)–1(b3) are high magnification TEM images revealing the changes in the surface from curved to flat and then to curved again as the irradiation dose increased at 25°C. The former process indicates the deformation of SiO_2_, which is attributed to beam-induced athermal activation of massive plastic flow and surface migration [23, 24]. The latter process is the formation of amorphous SiO_*x*_ NPs due to the desorption of O. The detailed process and low magnification images are shown in [Supplementary-material supplementary-material-1] (Supporting Information). In Figure 1(b4), a Si nucleus is observed in a SiO_*x*_ nanoparticle when the dose of electron irradiation is up to 2.64 × 10^8^ C m^−2^. This dose is approximately one order of magnitude higher than that at 400°C, indicating that heating can accelerate the formation of crystalline Si. However, the critical dose for SiO_*x*_ NP formation does not change noticeably, on the order of 10^6^ C m^−2^. Hence, the critical dose for SiO_*x*_ and Si NP formation will eventually overlap with increasing temperature. Figures 1(c1) and 1(c2) show that c-Si NPs appear directly under a dose of 1.0 × 10^7^ C m^−2^, without the observation of SiO_*x*_ NPs at 600°C. [Supplementary-material supplementary-material-1] (Supporting Information) shows the morphological changes at different dose rates. The critical dose for NP formation does not change significantly, which indicates that this process is dose-dependent. The Si (111) lattices are shown in Figure 1(c2). Figure 1(d) shows the changes of the Si *L*_2,3_ edge in the electron energy loss spectrum (EELS) with the increase of dose at 25°C. The edge at 99.8 eV is evidence of elemental Si, and it arises at a dose of 2.42 × 10^8^ C m^−2^, which is consistent with the observations of TEM images.

**Figure 2:**
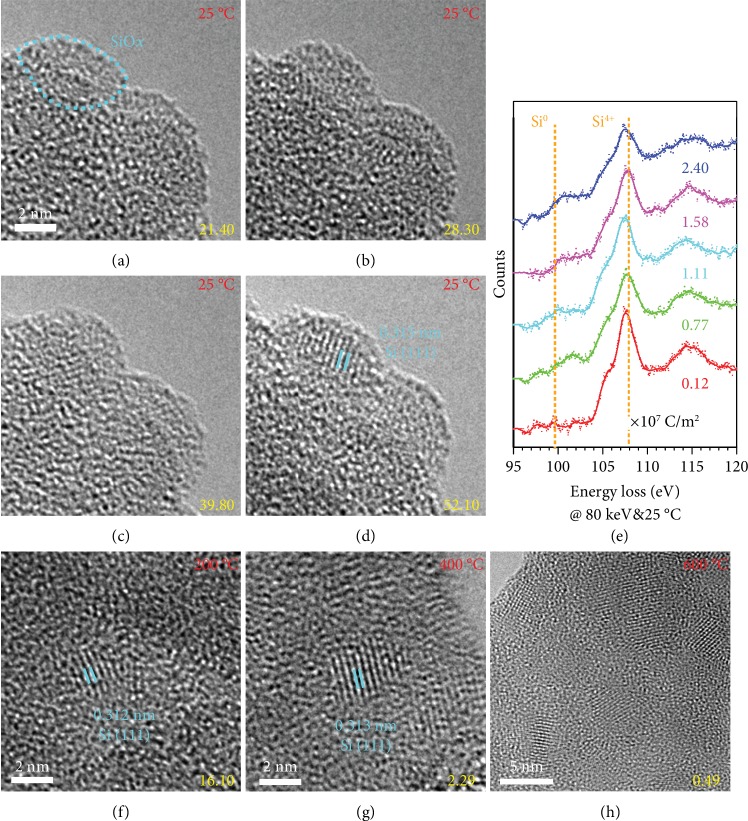
Formation of Si nanocrystals at different doses and temperatures under 80 keV electron irradiation. (a–d) Crystalline Si NP growth process as the dose increased at 25°C. The Si (111) lattice can be clearly seen in (d). (e) EELS of Si *L*_2,3_ edge at 25°C under different doses. (f–h) Formation of c-Si NPs at 200°C, 400°C, and 600°C with the corresponding dose. The unit of the dose is 10^6^ C m^−2^.

To investigate the influence of electron beam energy on the growth process of c-Si NPs, amorphous SiO_2_ was irradiated under 80 keV at different temperatures.  Figure 2(a)–2(d) shows the formation of c-Si NPs at 25°C. Amorphous SiO_*x*_ NPs can be observed at the surface highlighted by the cyan dashed oval in Figure 2(a). The critical dose for the formation of SiO_*x*_ NPs is approximately on the order of 10^5^ C m^−2^ and is independent of temperature, as shown in Figures [Supplementary-material supplementary-material-1] and [Supplementary-material supplementary-material-1] (Supporting Information). The c-Si is formed at a dose of 5.21 × 10^7^ C m^−2^, and the Si (111) lattice fringes are represented in Figure 2(d). These two critical doses are both approximately one order of magnitude lower than that under 300 keV electron irradiation. The EELS in Figure 2(e) confirms the formation of elemental Si when the dose increases to ~10^7^ C m^−2^. As the temperature increases, the critical dose for growth of c-Si NPs decreases (Figure 2(f)–2(h)), the same as in the case under 300 keV irradiation. The direct formation of Si NPs is also observed under 80 keV irradiation at 600°C (Figures 2(h) and [Supplementary-material supplementary-material-1], Supporting Information). These results show that the sensitivity of SiO_2_ for the fabrication of c-Si is higher under low energy electron irradiation at high temperature. Our findings are different from those proposed by Du et al., who believe that the formation of c-Si NPs results from the elastic displacement of Si atoms and that the threshold energy is 150.2 keV [12]. Hence, the c-Si NP growth mechanism cannot be the elastic displacement of Si atoms.

**Figure 3:**
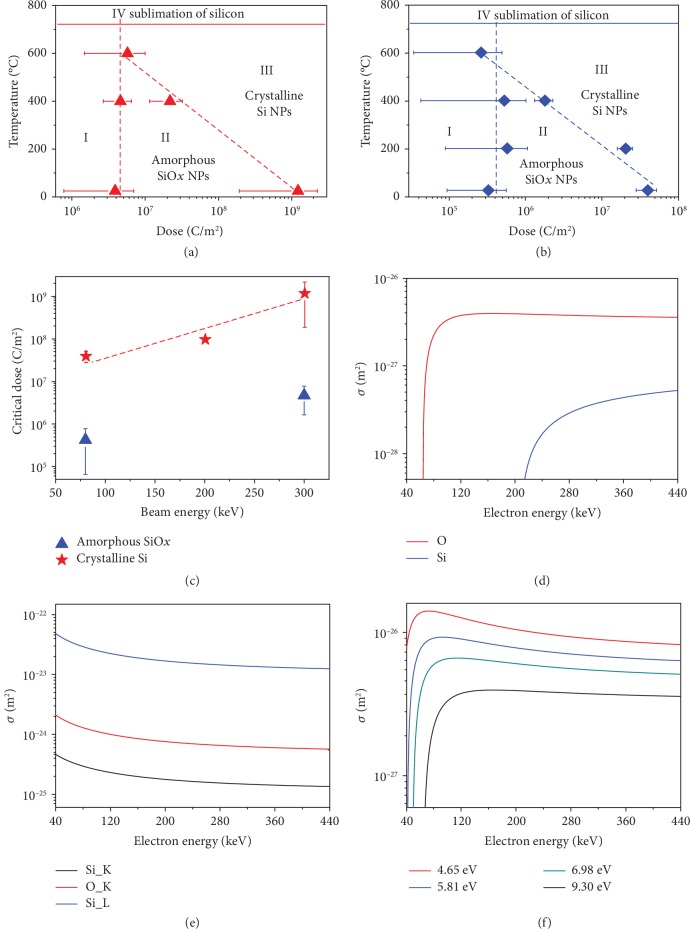
Phase diagram and electron scattering cross section. (a, b) Phase change during the growth process of crystalline Si NPs under 300 keV and 80 keV irradiation. Triangles and diamonds indicate the critical dose at different temperatures. The dashed lines correspond to linear fitting. (c) Critical dose for the formation of c-Si NPs at 25°C (red stars) and amorphous SiO_*x*_ NPs (blue triangles) under different beam energies. The dashed line is the linear fitting, and the data at 200 keV is reported by Du et al. [12]. (d, e) Elastic and inelastic electron scattering cross sections at different energies. (f) Elastic cross sections of O at different displacement threshold energies.

To elucidate the mechanism behind the growth process, we create phase diagrams for temperature and dose with different electron energies (Figures 3(a) and 3(b)). The phase diagram can be divided into four parts. Part I indicates the deformation of SiO_2_ under low-dose irradiation, which has been discussed in other works [23, 24]. Parts II and III represent the formation of amorphous SiO_*x*_ and crystalline Si NPs. In Figures 3(a) and 3(b), the critical dose of SiO_*x*_ NP formation is temperature insensitive, implying that this process is athermal. The critical dose is averaged for different temperatures and plotted in Figure 3(c), which indicates that the growth rate of SiO_*x*_ NPs is faster under low electron beam energy irradiation. However, the critical dose of c-Si NP formation decreases exponentially with increasing temperature. The relationships between the critical dose and temperature are acquired by linear fitting in Figures 3(a) and 3(b):

(1) $$\begin{array}{c} D=1.43\times 10^{9}e^{T/-104.09}\text{ C }{\text{m}}^{-2}\left(300\text{ keV}\right), \end{array}$$

(2) $$\begin{array}{c} D=8.02\times 10^{7}e^{T/-105.96}\text{ C }{\text{m}}^{-2}\left(80\text{ keV}\right), \end{array}$$

where *T* is the temperature at °C. The slopes in Figures 3(a) and 3(b) (coefficients in Equations (1) and (2)) are the same, indicating that the influence of temperature and electron beam energy is uncorrelated. There are intersections between the two critical doses at approximately 600°C, above which direct formation of c-Si NPs is observed. The critical dose of crystalline Si formation at room temperature under different electron beam energy irradiation is shown in Figure 3(c). The influence of electron beam energy on the critical dose is estimated by linear fitting in Figure 3(c) as follows:

(3) $$\begin{array}{c} D=8.92\times 10^{6}e^{E/65.62}\text{ C }{\text{m}}^{-2}, \end{array}$$

where *E* is the electron beam energy in keV. Because of the independence between temperature and electron beam energy, we can simply combine Equations (1), (2), and (3) to obtain the relationship between the critical dose and temperature and electron beam energy:

(4) $$\begin{array}{c} D=1.66\times 10^{7}e^{\left(E/65.62\right)-\left(T/105.03\right)}\text{ C }{\text{m}}^{-2},\;T\leq 600^{{}^{\circ}}\text{C}. \end{array}$$

Equation (4) implies that there are two types of mechanisms behind the growth process. One is electron irradiation-induced reduction of SiO_2_, which plays a part during the whole growth process (I➔II➔III). The dissolution of SiO_2_ can be induced by elastic sputtering and/or inelastic ionization. As discussed above, O is more easily sputtered, and in Figure 3(d), the elastic scattering cross section of O is approximately one order of magnitude larger than that of Si. The cross section is calculated with the Mckinley-Feshbach equation [15]. The core electron ionization can also result in desorption of O with the cross section:

(5) $$\begin{array}{c} \sigma_{O}=\sum \sigma_{\text{Core}}\times f_{O}\times P\left(t_{c}\right), \end{array}$$

where *σ*_Core_ is the core electron ionization cross section, *f*_*O*_ is the fraction of interatomic Auger events that result in two holes localized in a bonding orbital, and *P*(*t*_*c*_) is the probability that two holes are localized in the orbital at time *t*_*c*_ after being created in a surface bond orbital at time *t* = 0 [25]. The *σ*_*O*_ for SiO_2_ is 10^−26^–10^−25^ m^2^ when the electron beam energy is 150–3000 eV [25–28]. We assume that *f*_*O*_ and *P*(*t*_*c*_) remain constant with increasing electron beam energy because they are properties of SiO_2_. The core electron ionization cross section is acquired by Bote's analytical formulas and shown in Figure 3(e) and [Supplementary-material supplementary-material-1] (Supporting Information) [29]. The ionization cross section at 80–300 keV is approximately one-tenth of that at 150–3000 eV. Hence, the O desorption cross section at 80–300 keV is approximately 10^−27^–10^−26^ m^2^, which is comparable with the elastic sputtering cross section of O (Figure 3(d)). However, the creation of O vacancies leads to dangling bonds in Si atoms, and these unpaired electrons provide a channel for fast charge-transfer screening, which reduces the hole-hole correlation energy and lowers the lifetime of localized holes. Intuitively, an increase in the O vacancy density causes a decrease in *P*(*t*_*c*_) in Equation (5); therefore, the desorption of O by core electron ionization will be greatly suppressed. This result indicates that both elastic and inelastic scattering is responsible for O desorption at the beginning of the process, and then elastic scattering dominates with the continuous generation of O vacancies, which means that the formation of c-Si NPs is mainly driven by the elastic displacement of O. However, the critical dose reduces with decreasing electron beam energy, and the cross section does not change noticeably in Figure 3(d). The generation of O vacancies can cause the remaining O atoms to be more easily sputtered, hence reducing the *T*_*d*_. The cross section for O with different *T*_*d*_ is shown in Figure 3(f), in which a decrease of the cross section can be observed with an increase of electron beam energy. Therefore, the elastic displacement of O is responsible for c-Si formation.

**Figure 4:**
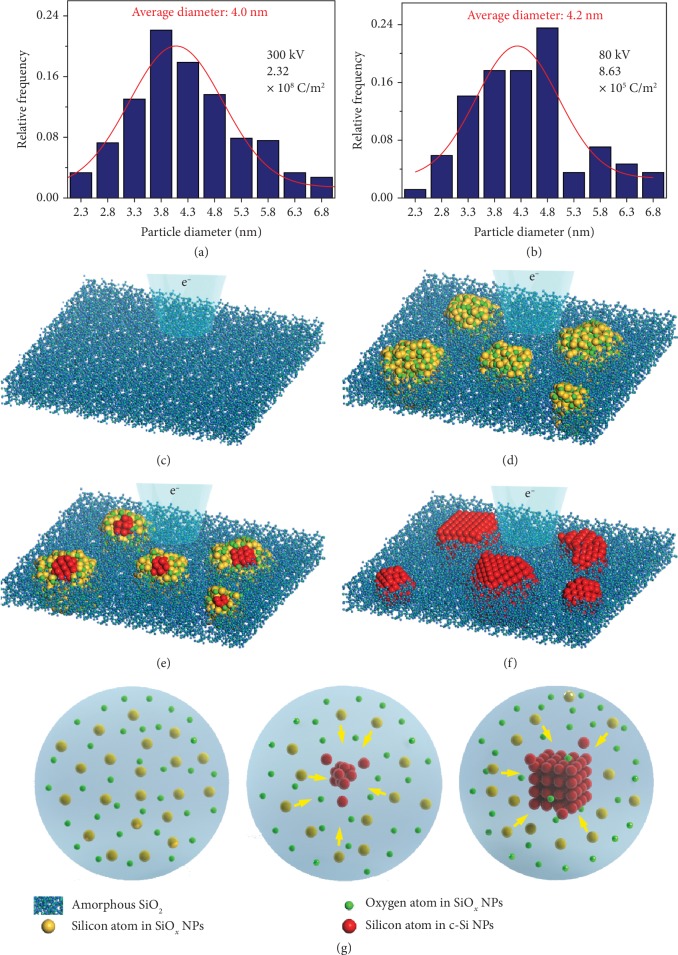
Equivalent diameter distribution and schematic diagram of the growth process of c-Si NPs. (a, b) Si NP size under 300 keV and 80 keV electron beam irradiation at 600°C. (c–g) Atomic models of the c-Si NP growth process. (a) The initial amorphous SiO_2_, (b) formation of amorphous SiO_*x*_ NPs under electron irradiation, (c) nucleation of crystalline Si in SiO_*x*_ NPs under further irradiation, and (d) complete transformation into c-Si NPs. (g) Details of the nucleation of c-Si from SiO_*x*_ NP.

The other mechanism shown in Equation (4) is temperature-dependent diffusion, which only influences the growth process of c-Si NPs (II➔III). Figures 4(a) and 4(b) show that the equivalent average diameter of c-Si NPs is approximately 4 nm under different electron energies at 600°C. The average diameter is slightly smaller at 400°C ([Supplementary-material supplementary-material-1], Supporting Information). The diffusing and condensing of Si atoms is essential for c-Si NP formation. However, without irradiation, silicon oxide is more energy favorable than crystalline silicon [26, 30]. SiO_2_ under high-intensity irradiation is an open and highly dissipative system. Therefore, the growth of c-Si NPs is a self-organization process rather than an equilibrium thermodynamic process from the perspective of energy [31, 32]. This ordering phenomenon has also been observed in the transformation of carbon onions to diamonds under electron irradiation [33]. The ratio of thermal-activated carbon atom jump rates across the interface between graphite (G) and diamond (D) obeys the relationship Γ_*G*‐*D*_/Γ_*D*‐*G*_ = exp(‐Δ*G*/*k*_*B*_*T*), where *ΔG* is the Gibbs free energy difference between diamond and graphite, *k_B_* is Boltzmann's constant, and *T* is temperature. Without irradiation, *ΔG* is positive and carbon atoms tend to diffuse from diamond to graphite. Apart from thermally activated jumps, ballistic jumps may lead to atom exchanges between graphite and diamond, when the system is under electron irradiation. Considering these ballistic jumps, the nonequilibrium effective free energy difference is expressed using Zaiser-Banhart's equation [33]:

(6) $$\begin{array}{c} \Delta G_{\text{eff}}=\Delta G-k_{B}T\ln\frac{1+\varphi \sigma_{G}/r_{\text{th}}}{1+\varphi \sigma_{D}/r_{\text{th}}}, \end{array}$$

where *r*_th_ is the thermal jump rate of atoms across the interface, *φ* is the irradiation flux, and *σ*_*G*_ and *σ*_*D*_ are elastic displacement cross sections in graphite and diamond, respectively. The equation is defined to describe the phase stability under irradiation [33]. When the system is under electron irradiation at not too high temperature (small *r*_th_, *φσ*_*G*_/*r*_th_>>1, *φσ*_*D*_/*r*_th_>>1), the nonequilibrium free energy of diamond is reduced by approximately *k*_*B*_*T* ln(*σ*_*G*_/*σ*_*D*_). Because *σ*_*G*_ is much larger than *σ*_*D*_, the effective free energy difference is reduced below zero, which means an inversion of phase stability [33]. In this work, the displacement cross section of SiO_*x*_ is larger than that of Si because of the much lower *T*_*d*_ (O). The ratio of the cross section is approximately 10 and ∞ when the electron beam energy is above 150.2 keV and between 64.0 and 150.2 keV, as shown in Figure 3(d). The effective free energy of c-Si is greatly reduced, and transformation from amorphous SiO_*x*_ NPs to c-Si NPs is activated. Intuitively, the high vacancy density in SiO_*x*_ produces many high-energy elemental Si atoms with strong diffusivity, and the low vacancy density makes the Si atoms in the c-Si stable with weak diffusivity.

Control experiments have been conducted to confirm that heating alone up to 600°C cannot induce the transformation from amorphous SiO_*x*_ NPs to c-Si NPs or even reduce defects in the as-formed c-Si NPs (Figures [Supplementary-material supplementary-material-1] and [Supplementary-material supplementary-material-1], Supporting Information). This temperature is below the Si NP crystallization temperature at approximately 800°C [34]. However, due to the high vapor pressure of Si, the sublimation temperature is lower than the crystallization temperature under an ultrahigh vacuum, and sublimation of Si is observed when the temperature is increased to above ~750°C (Figures 3(a) and 3(b) and Figures [Supplementary-material supplementary-material-1] and [Supplementary-material supplementary-material-1], Supporting Information) [19]. Moreover, the only dissociation of SiO_2_ is observed without the formation of SiO_*x*_ or Si NPs when SiO_2_ is irradiated at 900°C ([Supplementary-material supplementary-material-1], Supporting Information). Directly heating SiO_2_ up to 1000°C and even repeatedly heating SiO_2_ between 25°C and 1000°C cannot cause the growth of SiO_*x*_ and Si NPs ([Supplementary-material supplementary-material-1], Supporting Information). All these results reveal that electron irradiation is the key factor for c-Si NP formation, and the heating effects induced by irradiation are also excluded.

The growth process can be divided into two parts as shown in Figure 4(c)–4(f): formation of amorphous SiO_*x*_ and c-Si NPs. As discussed above, initially, the desorption of O is induced by elastic sputtering (O^0^) and core electron ionization (O^+^), resulting in the growth of silicon suboxide (Figure 4(d)). This process does not involve the thermal diffusion of atoms, so the critical dose is electron beam energy dependent but temperature independent. Continuous generation of O vacancies not only suppresses core electron ionization-induced desorption but also reduces the threshold energy of O, which increases the displacement cross section in SiO_*x*_ and reduces the effective free energy difference between Si and SiO_*x*_. Once the energy difference becomes negative, the Si atoms start to condense and c-Si NPs nucleate from the SiO_*x*_ NPs, as shown in Figures 4(e) and 4(g). With increasing doses, the amorphous SiO_*x*_ NPs completely transform into c-Si NPs. Equation (4) reveals that the critical dose for c-Si NP formation decreases when the temperature is increased, and the electron beam energy is reduced. From the energy perspective, increasing temperature and reducing electron beam energy can both lower the effective free energy of c-Si, leading to larger rates of Si atom diffusion from SiO_*x*_ to Si. Larger rates mean a short time for growth and a low integrated dose. When the temperature is above 600°C, the c-Si NPs grow so fast that amorphous SiO_*x*_ NPs cannot be observed in our experiments. It is worth noting that the constant under *E* in Equation (4) is the same as the threshold electron beam energy (64 keV) for O displacement in SiO_2_ [16]. This result may imply that to trigger the growth process, the electron beam energy should be larger than ~65.62 keV. Similarly, the constant under *T* may imply that the self-organization process only occurs above -105.03°C. Amorphization in crystalline Si under electron irradiation was observed at -248°C [35]. However, to confirm the physical meaning of these two constants, further detailed experiments should be carried out.

## 3. Conclusion

In summary, the formation of c-Si NPs from amorphous SiO_*x*_ NPs is a self-organization process induced by the elastic displacement of O. The high displacement cross section of O in SiO_*x*_ significantly reduces the effective free energy of c-Si, causing the phase stability inversion between c-Si and amorphous oxide and thus promoting the growth of c-Si NPs. Quantitative experiments reveal that the critical dose for c-Si NP formation decreases exponentially with increasing temperature and reducing electron beam energy. The exposure speed during direct electron writing can be enhanced by three orders of magnitude under 80 keV irradiation at 600°C. The formation of amorphous SiO_*x*_ NPs is attributed to O desorption induced by elastic sputtering and core electron ionization, which will be suppressed by a high density of O vacancies. The critical dose for SiO_*x*_ NP formation is temperature independent and decreases with high scattering cross sections under low electron beam energy. Our work reveals the detailed mechanism and quantitative conditions of the fabrication process and provides valuable information for direct electron beam writing for the fabrication of Si into SiO_2_.

## 4. Materials and Methods

### 4.1. Preparation of Samples

The amorphous SiO_2_ was purchased from Xianfeng Nano Materials Co., Ltd. Firstly, the power was dispersed in deionized water. After sonication for 30 min, a drop (~10 *μ*L) of the suspension was placed at the center of the heating chip (Aduro 100, Protochips Inc., and Wildfire, DENSsolutions Inc.) and dried under ambient conditions.

### 4.2. In Situ TEM Observation

The growth process is conducted in a Cs-corrected transmission electron microscope (FEI Titan 80-300) with a beam current density of 0.31–5.4 × 10^4^ A m^−2^ at 300 keV and 1.28 × 10^4^ A m^−2^ at 80 keV.

## Supplementary Materials

Supplementary Materials Figure S1: morphological changes corresponding to Figure 1 at 400°C under different doses. Figure S2: morphological deformation of SiO_2_ and formation of SiO*_x_* NPs at 25°C under different doses. Figure S3: morphological changes at 600°C under different dose rates when electron energy is 300 keV. Figure S4: 80 keV electron beam irradiation effects at 25°C. Figure S5: morphological deformation and NP formation at different temperatures under 80 keV. Figure S6: inelastic cross section of the Si L shell for low energy electron. Figure S7: crystalline Si NP equivalent diameter distribution. Figure S8: heating effects on as-formed SiO*_x_* NPs without electron irradiation. Figure S9: heating effects for as-formed c-Si NPs without electron irradiation. Figure S10: sublimation of as-formed crystalline Si NPs at high temperature. Figure S11: heating effects when temperature was increased above 800°C. Figure S12: fast dissolution of SiO_2_ at 900°C under 300 keV irradiation without c-Si NP formation. Figure S13: heating effects for SiO_2_ without electron irradiation.
