# Supplementary material for: In Situ Observation of Crystalline Silicon Growth from SiO2 at Atomic Scale
Source: Research (Wash D C). 2019 Oct 30;2019:3289247. doi: 10.34133/2019/3289247 (PMC6944520; doi:10.34133/2019/3289247)
Supplement: Supplementary Materials — Figure S1: morphological changes corresponding to Figure 1 at 400°C under different doses. Figure S2: morphological deformation of SiO2 and formation of SiOx NPs at 25°C under different doses. Figure S3: morphological changes at 600°C under different dose rates when electron energy is 300 keV. Figure S4: 80 keV electron beam irradiation effects at 25°C. Figure S5: morphological deformation and NP formation at different temperatures under 80 keV. Figure S6: inelastic cross section of the Si L shell for low energy electron. Figure S7: crystalline Si NP equivalent diameter distribution. Figure S8: heating effects on as-formed SiOx NPs without electron irradiation. Figure S9: heating effects for as-formed c-Si NPs without electron irradiation. Figure S10: sublimation of as-formed crystalline Si NPs at high temperature. Figure S11: heating effects when temperature was increased above 800°C. Figure S12: fast dissolution of SiO2 at 900°C under 300 keV irradiation without c-Si NP formation. Figure S13: heating effects for SiO2 without electron irradiation. [file 3289247.f1.docx]

Supplementary Materials

In Situ Observation of Crystalline Silicon Growth from SiO_2_ at Atomic Scale

**Kaihao Yu^1,†^, Tao Xu^1,†^, Xing Wu^2,*^, Wen Wang^1^, Hui Zhang^1^, Qiubo Zhang^1^, Luping Tang^1^, Litao Sun^1,*^**

^1^ SEU-FEI Nano-Pico Center, Key Laboratory of MEMS of Ministry of Education,

School of Electronic Science and Engineering, Southeast University, Nanjing 210096,

China.

^2^ Shanghai Key Laboratory of Multidimensional Information Processing,

Department of Electronic Engineering, East China Normal University,

Shanghai 200241, China.

^*^Correspondence should be addressed to Litao Sun; [slt@seu.edu.cn](mailto:slt@seu.edu.cn) and Xing Wu; [xwu@ee.ecnu.edu.cn](mailto:xwu@ee.ecnu.edu.cn).

^†^ These authors contributed equally to this work.


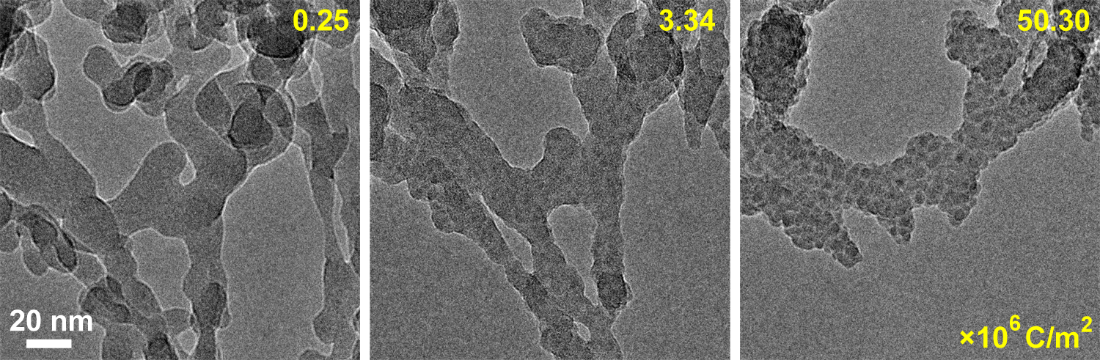


FIGURE S1: *Morphological changes corresponding to Figure 1 at 400 °C under different doses.*


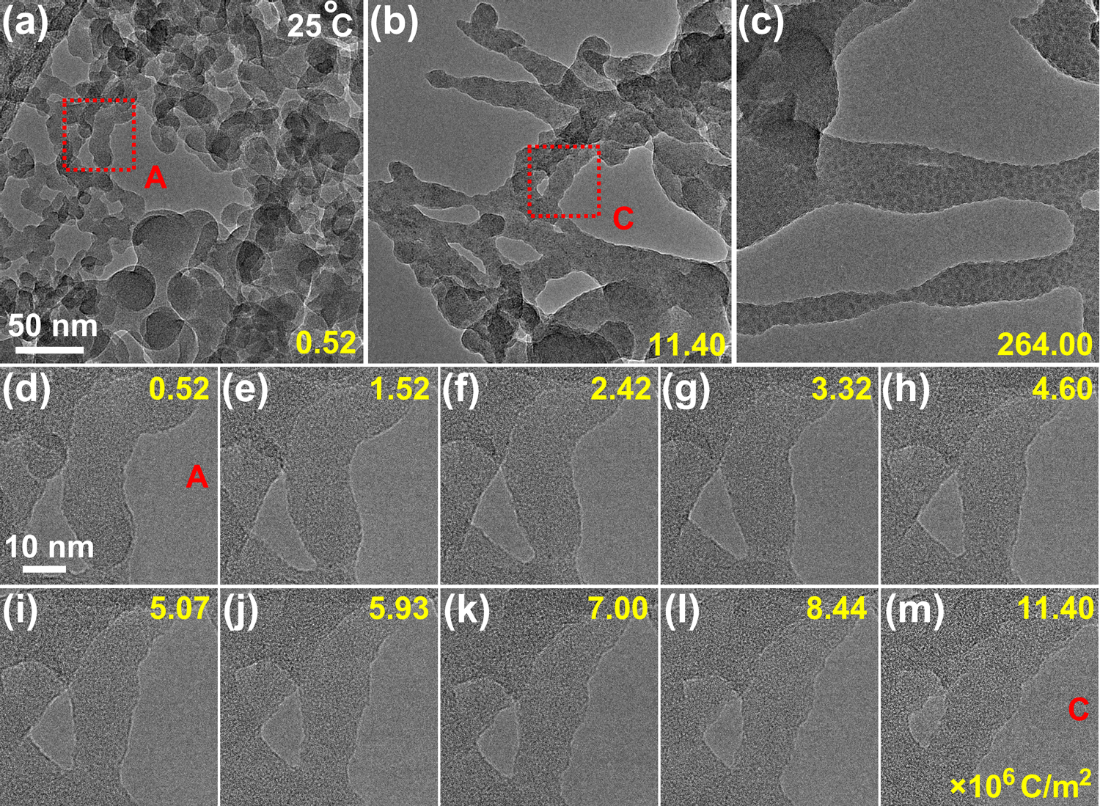


FIGURE S2: *Morphological deformation of SiO_2_ and formation of SiO_x_ NPs at 25 °C under different doses.* (a)–(c) Low magnification images show the shape deformation and particle formation. (d)–(m) Details of the SiO_x_ NPs formation process corresponding to FigureS 1(a)–(d).


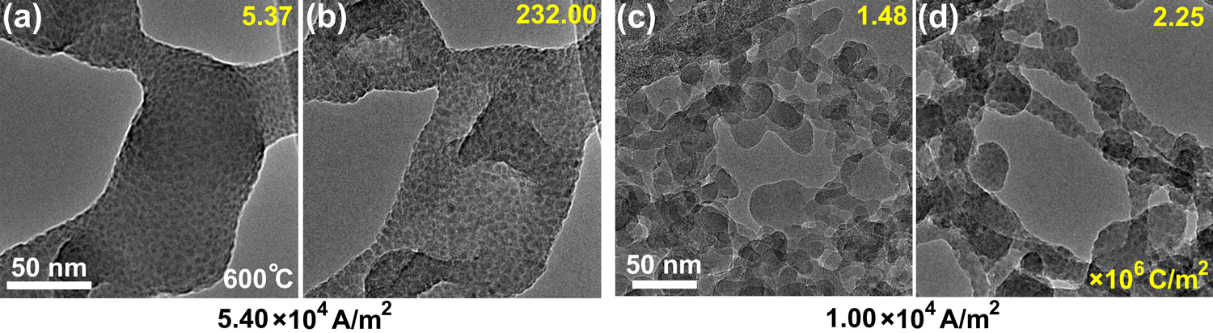


FIGURE S3: *Morphological changes corresponding to Figure 1(j)–(m) at 600 °C under different dose rates.*


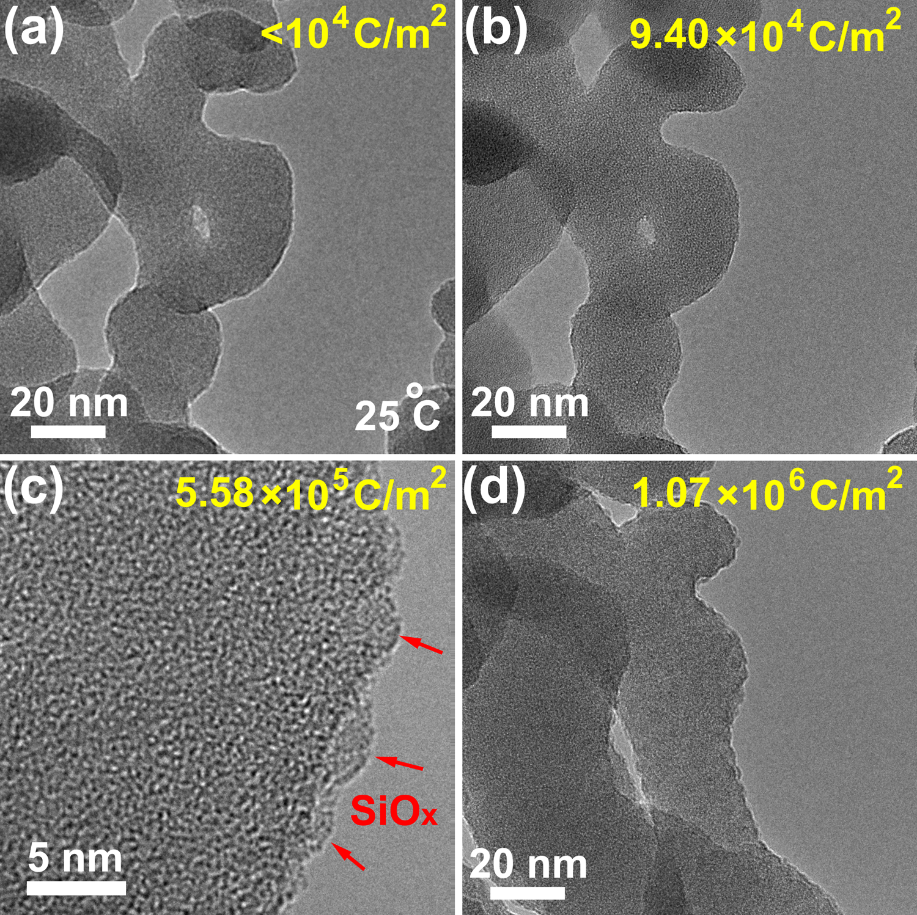


FIGURE S4: *80 keV electron beam irradiation effects at 25 °C.* (a)–(d) SiO_2_ deformation and SiO_x_ NPs formation under different doses.


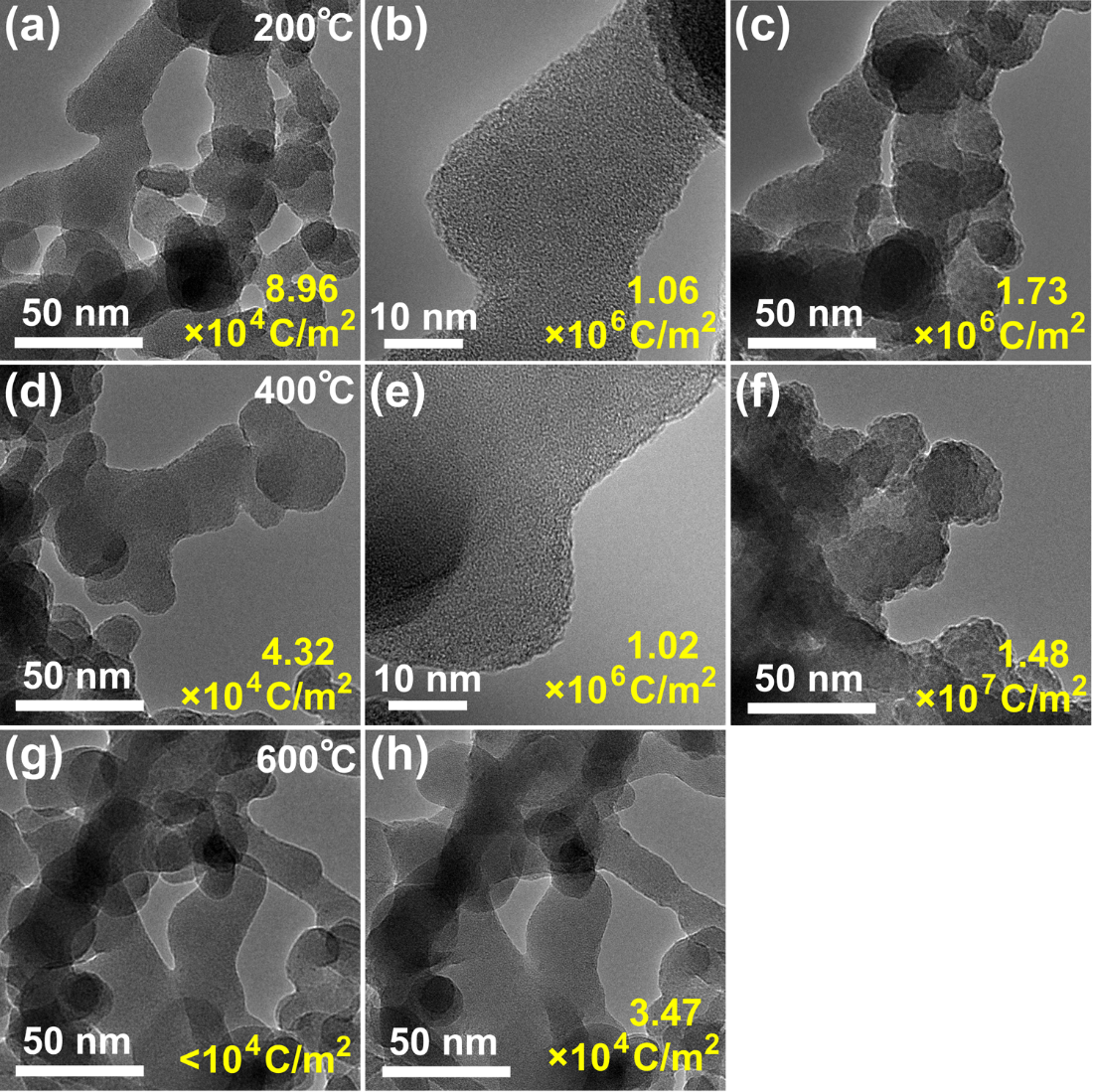


FIGURE S5: *Morphological deformation and NPs formation at different temperatures under 80 keV*. (a)–(c) Deformation of SiO_2_ at 200 °C, (d)–(f) at 400 °C, (g)–(h) and at 600 °C.


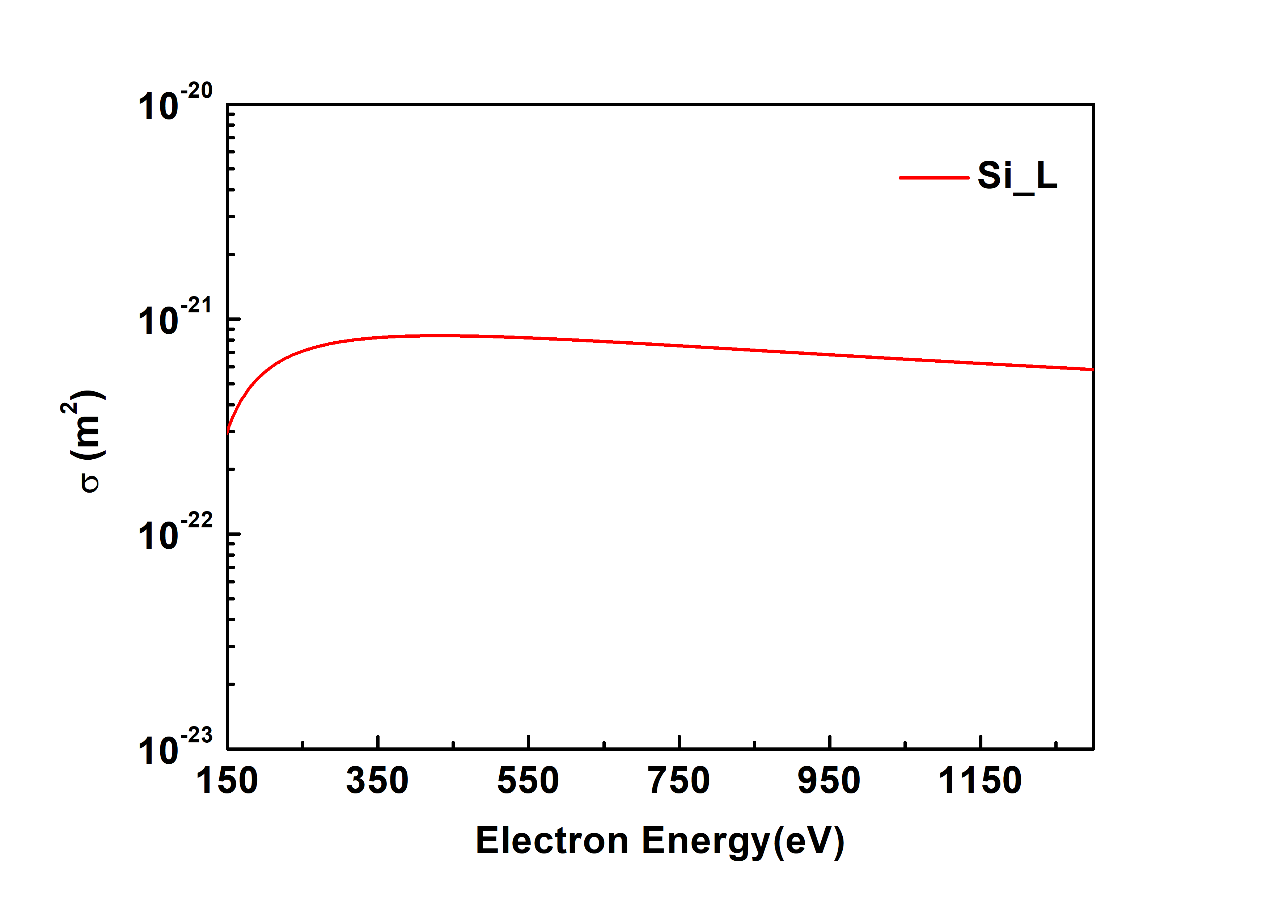


FIGURE S6: *Inelastic cross section of the Si L shell for low energy electron.*


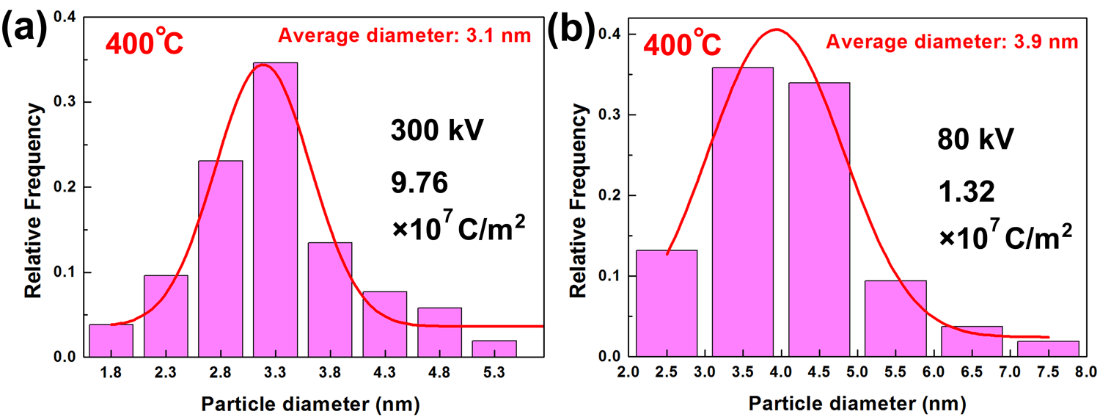


FIGURE S7: *Crystalline Si NPs equivalent diameter distribution.* (a), (b) c-Si NPs size under 300 keV and 80 keV electron beam irradiation at 400 °C.


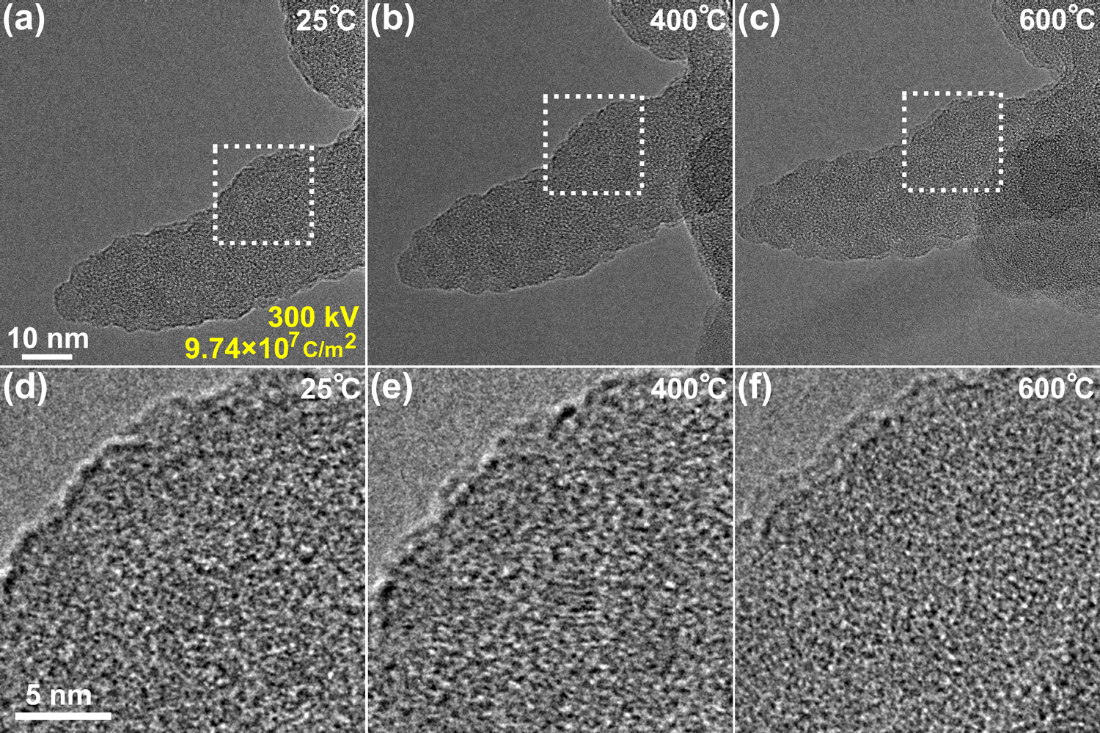


FIGURE S8: *Heating effects on as-formed SiO_x_ NPs without electron irradiation.* (a)–(c) Low magnification images and (d)–(f) high resolution images showing no crystalline Si NPs are formed.


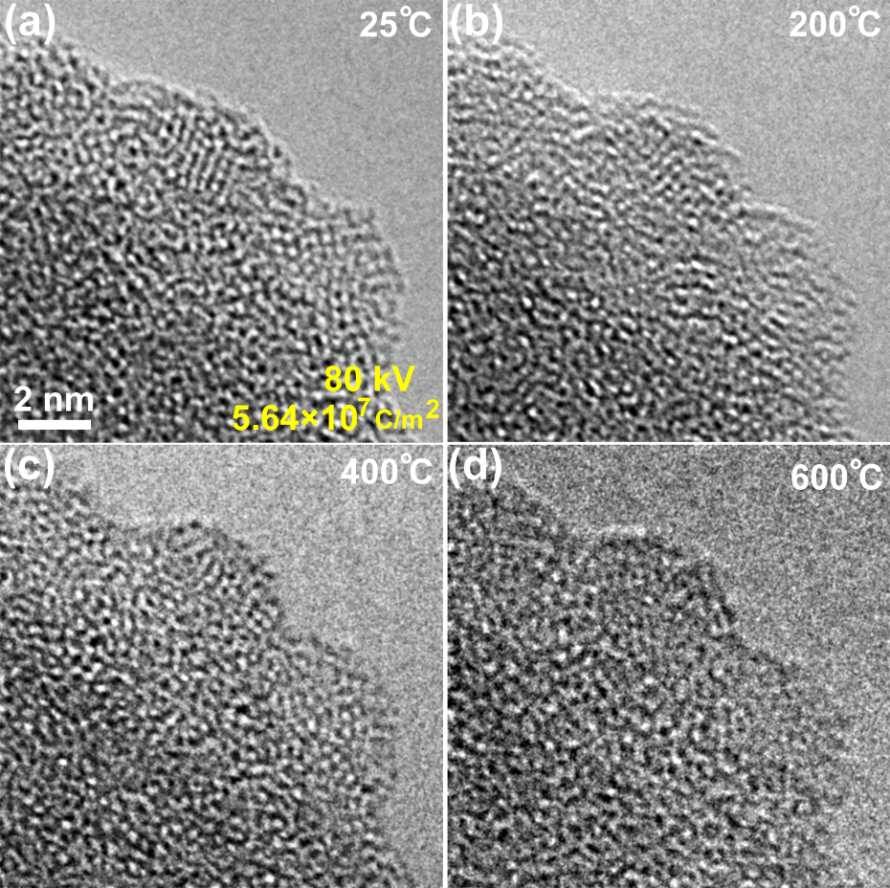


FIGURE S9: *Heating effects for as-formed c-Si NPs without electron irradiation*. (a) Electron beam-induced defective crystalline Si NPs at 25 °C. (b)–(d) Annealing of the NPs at different temperatures indicating that defects are not reduced.


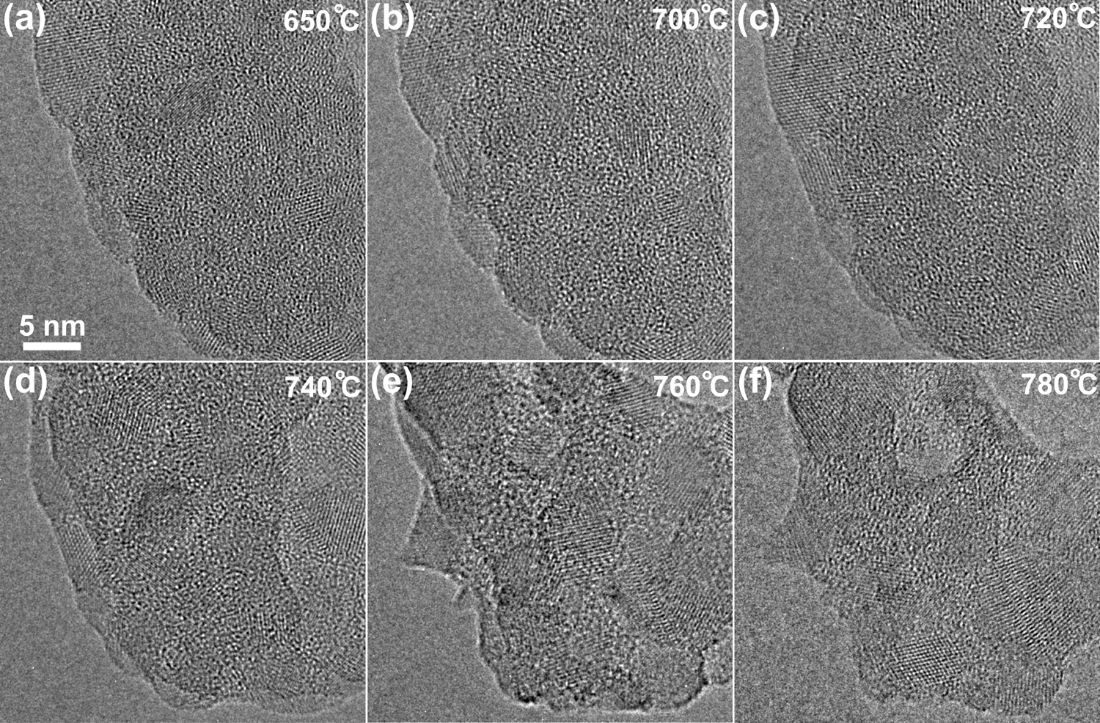


FIGURE S10: *Sublimation of as-formed crystalline Si NPs at high temperature.* (a), (b) No apparent sublimation below 700 °C. (c)–(f) Si starting to sublimation when the temperature increased above 720 °C.


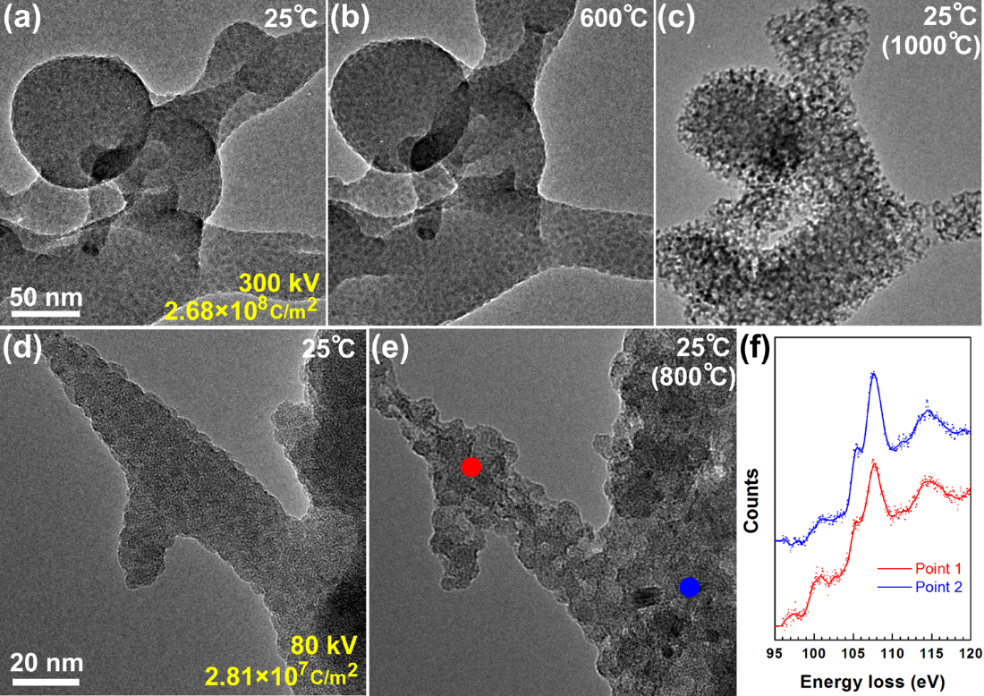


FIGURE S11: *Heating effects when temperature was increased above 800 °C*. (a) SiO_x_ NPs formed at 25 °C under 300 keV electron irradiation. (b) Annealing at 600 °C showing no apparent change. (c) Cooling down to 25 °C after heating to 1000 °C, showing sublimation of the NPs. (d)–(e) 80 keV electron beam-induced formation of SiO_x_ NPs at 25 °C and subsequent sublimation at 800 °C. (f) EELS of the Si *L_2,3_* edge after sublimation. Most of the remains are SiO_2_, and there are still some elemental Si. Irradiation is not introduced during sublimation.


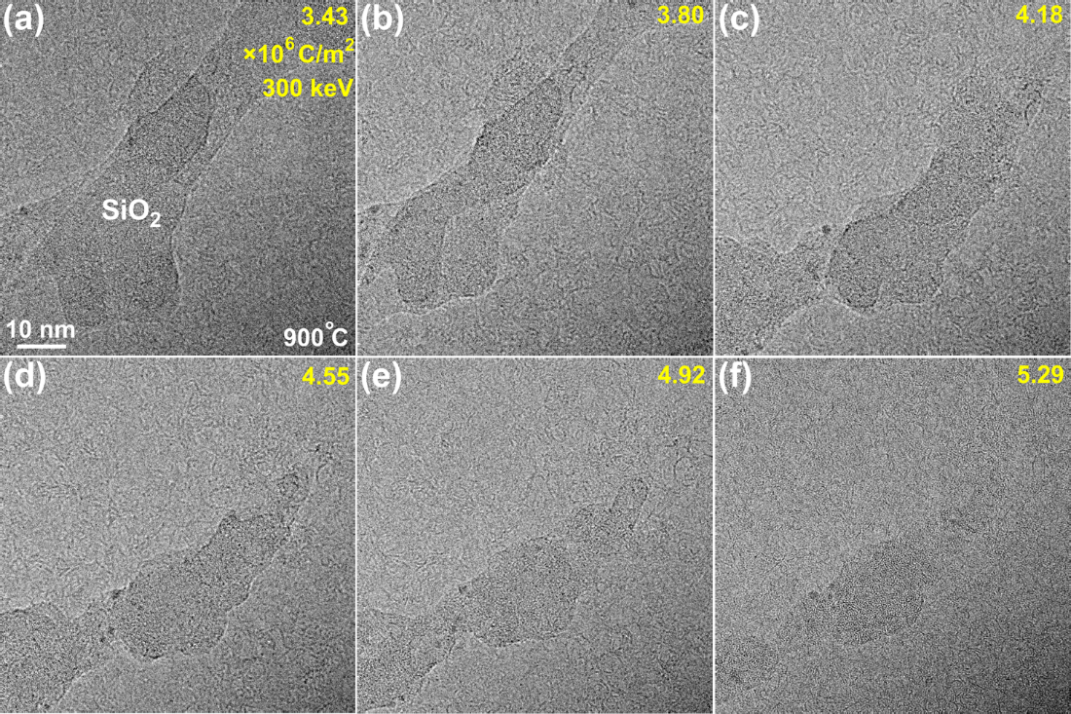


FIGURE S12: *Fast dissolution of SiO_2_ at 900 °C under 300 keV irradiation without c-Si NPs formation.*


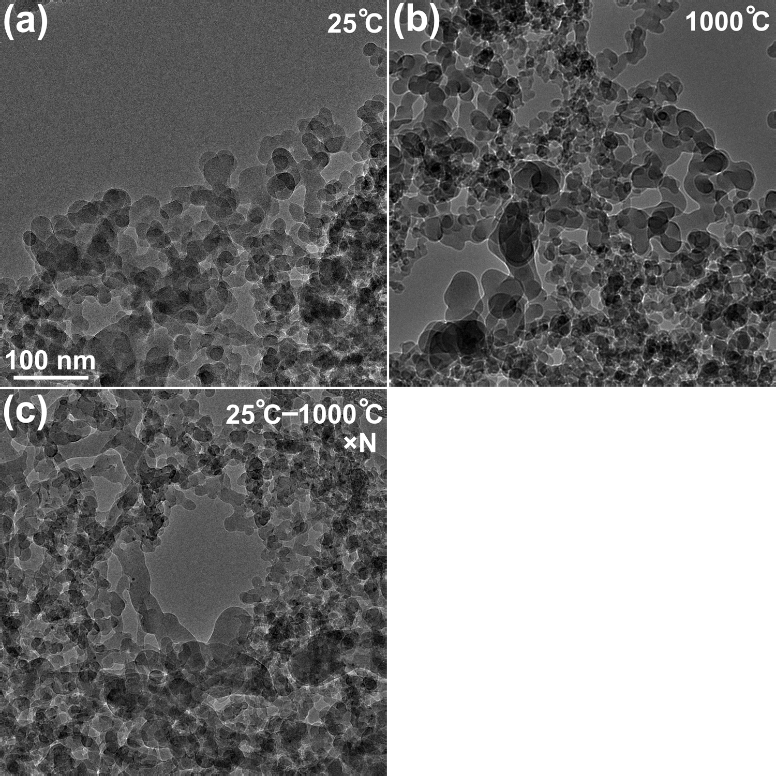


FIGURE S13: *Heating effects for SiO_2_ without electron irradiation.* (a) Original SiO_2_ without electron irradiation. (b) SiO_2_ heated to 1000 °C without electron irradiation. (c) SiO_2_ repeatedly heated between 25 °C and 1000 °C without electron irradiation. No apparent changes can be observed.
